# The Anatomy and Diseases of the Breast

**Published:** 1845-10

**Authors:** 


*The Anatomy and Diseases of the Breast. With numerous plates.* By SIR ASTLEY COOPER, Bart., F. R. S., Sergeant Surgeon to his Majesty; Consulting Surgeon of Guy's Hospital; Lecturer on Anatomy and Surgery, &c. &c. *To which are added his various Surgical Papers, now first published in a collected form.* Royal 8vo., pp. 188. Philadelphia, 1845.

This volume completes the splendid edition by Messrs. Lea & Blanchard of the works of the distinguished Surgeon; for which they merit the thanks of the profession. It was well thought of when they they determined to collect also his various surgical papers, which have never previously been brought together. "This volume," they remark, "with those already published in this country on the subject of Hernia, the Structure and Diseases of the Testis and Thymus Gland, Fractures and Dislocations of the Joints, and Lectures on Surgery, edited by Tyrrell, making five volumes in all, complete the valuable series of the works of Sir Astley Cooper. The present corresponds in size with the other *illustrated* works of the author. Great care has been taken in the reduction and execution of the plates in lithography—and the publishers trust the figures will be found to be faithful copies of the larger and more expensive originals. The sources from which the surgical papers and contributions have been collected, are stated at the head of each article, and were pointed out in the list appended to a slight biography of Sir Astley, published in the last edition of his work on 'Dislocations and Fractures.'" "The publishers," they add, "feel that they have fully redeemed the pledge given by them, of presenting the productions of the great modern English Surgeon in a form which, at the same time that it places them within the reach of all members of the profession, is worthy of the fame of the illustrious author."

We apprehend that the series will be sought after by all who are familiar—and who is not—with the pre-eminent merits of Sir Astley as an Anatomist and Surgeon.

*Outlines of the Arteries: with short Descriptions. Designed for the use of Medical Students.* By John Neill, A. M., M. D., Prosector in the University of Pennsylvania; Physician to Wills' Hospital; Lecturer on Anatomy, etc. Philadelphia: Barrington and Haswell. 1845.

The above is the title of a very neat and useful little work, prepared, as the author informs us, "at the request of several students, to furnish them with an easy, yet accurate, mode of learning the arteries." It is true, there is no lack of works descriptive of this branch of anatomical science; they abound, indeed, in every country where medicine is taught. But the splendid and highly finished plates of the French and English, and—we are happy to be able to add—American anatomists, are far too costly, if not too elaborate, for the beginner.

However proper, and indeed necessary, it may be for the student of Anatomy to become well acquainted with the exact position of the blood vessels in relation to other parts of the body, yet his elementary knowledge of them must regard the different portions of the circulatory system as dependent chiefly upon each other: he must, for example, first learn what branches spring from this or that particular artery, and how they are distributed. Now, these are just the points which we think are admirably exhibited in the little work before us, which is very creditable, both in the drawing and lithography. Its chief excellency, however, is its *mode of reference*, in which it differs from the usual method of numbering the different vessels, and referring to these numbers in the accompanying explanation. In the present instance, each artery has its own name legibly printed upon it, so that the student can, at a glance perceive both the position of the vessel and its name, without the tediousness of a constant reference.

The author designs, we understand, to follow up this work with "*Outlines of the Nerves*," arranged after a similar method. *

